# Timing of Antithrombotic Secondary Prevention in Patients with Intracranial Hemorrhage after Stroke Thrombolysis and Thrombectomy

**DOI:** 10.3390/jcm12082771

**Published:** 2023-04-07

**Authors:** Giuseppe Reale, Pietro Caliandro, Tiago T. P. Moreira, Håkan Almqvist, Silvia Giovannini, David Grannas, Maria Ioanna Kotopouli, Andrea Laurienzo, Harald Löfberg, Marco Moci, Sebastian Sköldblom, Iacopo Valente, Aurelia Zauli, Staffan Holmin, Michael V. Mazya

**Affiliations:** 1 UOC Neuroriabilitazione ad Alta Intensità (Cod. 75), Fondazione Policlinico Universitario A. Gemelli IRCCS, 00168 Rome, Italy; 2 Department of Neurosciences, Università Cattolica del Sacro Cuore, 00168 Rome, Italy; 3 UOC Neurologia, Fondazione Policlinico Universitario A. Gemelli IRCCS, 00168 Rome, Italy; 4 Department of Neurology, Karolinska University Hospital, 17177 Stockholm, Sweden; 5 Department of Clinical Neuroscience, Karolinska Institutet, 17177 Stockholm, Sweden; 6 Department of Radiology, Capio St Göran’s Hospital, 11219 Stockholm, Sweden; 7 UOC Riabilitazione 2, Fondazione Policlinico Universitario A. Gemelli IRCCS, 00168 Rome, Italy; 8 Division of Biostatistics, Institute of Environmental Medicine, Karolinska Institutet, 17177 Stockholm, Sweden; 9 P.O. A. Cardarelli S.C. Neurologia-Stroke Unit, 86100 Campobasso, Italy; 10 Department of Internal Medicine, Nyköping Hospital, 61139 Nyköping, Sweden; 11 Division of Oncology, Karolinska University Hospital, 17177 Stockholm, Sweden; 12 UOC Diagnostica Per Immagini, Fondazione Policlinico Universitario A. Gemelli IRCCS, 00168 Rome, Italy; 13 Department of Neuroradiology, Karolinska University Hospital, 17177 Stockholm, Sweden

**Keywords:** ischemic stroke, thrombolysis, thrombectomy, aspirin, anticoagulants, secondary prevention, cerebral hemorrhage, hemorrhagic infarction

## Abstract

In patients with acute ischemic stroke, hemorrhagic transformation (HT) of infarcted tissue frequently occurs after reperfusion treatment. We aimed to assess whether HT and its severity influences the start of secondary prevention therapy and increases the risk of stroke recurrence. In this retrospective dual-center study, we recruited ischemic stroke patients treated with thrombolysis, thrombectomy or both. Our primary outcome was the time between revascularization and the start of any secondary prevention therapy. The secondary outcome was ischemic stroke recurrence within three months. We compared patients with vs. without HT and no (n = 653), minor (n = 158) and major (n = 51) HT patients using propensity score matching. The delay in the start of antithrombotics or anticoagulants was median 24 h in no HT, 26 h in minor HT and 39 h in major HT. No and minor HT patients had similar rates of any stroke recurrence (3.4% (all ischemic) vs. 2.5% (1.6% ischemic plus 0.9% hemorrhagic)). Major HT patients had a higher stroke recurrence at 7.8% (3.9% ischemic, 3.9% hemorrhagic), but this difference did not reach significance. A total of 22% of major HT patients did not start any antithrombotic treatment during the three-month follow-up. In conclusion, the presence of HT influences the timing of secondary prevention in ischemic stroke patients undergoing reperfusion treatments. Minor HT did not delay the start of antithrombotics or anticoagulants compared to no HT, with no significant difference in safety outcomes. Major HT patients remain a clinical challenge with both a delayed or lacking start of treatment. In this group, we did not see a higher rate of ischemic recurrence; however, this may have been censored by elevated early mortality. While not reaching statistical significance, hemorrhagic recurrence was somewhat more common in this group, warranting further study using larger datasets.

## 1. Introduction

Intravenous thrombolysis (IVT) and endovascular thrombectomy (EVT) are effective treatments for acute ischemic stroke. Hemorrhagic transformation of infarcted tissue (HT) commonly occurs after these treatments. This is commonly sub-classified into petechial hemorrhagic infarction (HI) and parenchymal hematoma (PH), the latter sometimes also occurring outside infarcted tissue and classified as remote PH (rPH) [1,2]. In patients undergoing imaging follow-up within 36 h from IVT, HI is seen in 10–11% and PH in 8–9% [3,4]. More HI occurs after EVT in the range of 19–30% and PH in 4–9% [5].

Intracranial hemorrhage (ICH) detected on routine early imaging follow-up after IVT or EVT may influence the start of antithrombotic and anticoagulant drugs for secondary stroke prevention. A multi-center observational study of stroke in atrial fibrillation showed that patients with and without hemorrhagic transformation started oral anticoagulants after a mean 23 vs. 12 days after stroke [6]. A large series of 150 patients with HI, diagnosed by MRI within five days after ischemic stroke, reported that 74% of patients were already on antithrombotic therapy: 53% on antiplatelets and 21% on warfarin. After HI detection, the great majority of patients continued the antithrombotic therapy, and this was not associated with neurological deterioration but with a lower frequency of the composite outcomes of neurological deterioration, vascular events and death (1.6% vs. 11.1%) at one month. Follow-up imaging did not show any HI worsening, despite the use of antiplatelets, anticoagulants or discontinuation [7].

We have been unable to find studies or guideline recommendations addressing timing of antiplatelets or anticoagulants in patients with HT after IVT, EVT or both. Moreover, it is unknown if a delay of secondary prevention due to HT is associated with increased early stroke recurrence in patients who received acute reperfusion treatment. 

Our primary aim was to assess if intracranial hemorrhage after reperfusion treatment is associated with a delayed start of antithrombotics and anticoagulants for secondary stroke prevention among stroke patients treated with IVT, EVT or their combination.

Secondary aims were (a) to evaluate if such a delay differs between patients with major, minor and no hemorrhage, and (b) to assess differences in stroke recurrence within three months between patients with major, minor and no hemorrhage.

## 2. Materials and Methods

This is a retrospective matched control dual-center study, pooling patient data collected at the Fondazione Policlinico Universitario A. Gemelli IRCCS in Rome, Italy and the Karolinska University Hospital in Stockholm, Sweden. The dataset from Rome consisted of consecutive patients with ischemic stroke receiving IVT, EVT or their combination in 2016–2019. The dataset from Stockholm consisted of consecutive patients receiving IVT, EVT or both in 2013–2016. The reason for choosing two different timeframes, after a thorough review of similarities in practice between the centers, was also the availability of a suitable, detailed dataset from Stockholm from those years. This study was approved by both the Stockholm and Rome Ethics Review Board. Written consent was obtained. The corresponding author has full access to all the data in this study and takes responsibility for their integrity and analysis.

We collected baseline clinical characteristics: age, sex, hypertension, atrial fibrillation (AF, previous or new diagnosis while in hospital), previous antithrombotic therapy, NIHSS, onset-to-IVT and onset to artery puncture times, CHA₂DS₂-VASc and HAS-BLED scores.

We classified post-treatment ICH on CT within 36 h using Heidelberg and SITS-MOST definitions [1,2]. Two independent authors analyzed scans for hemorrhage classification. Disagreement was resolved through consensus during a joint reading session, and, whenever an agreement was not reached, the final decision was made by invitation of a third independent colleague. ICH was classified as petechial hemorrhagic infarction (HI), parenchymal hematoma (PH), remote parenchymal hematoma (rPH), subarachnoid hemorrhage (SAH) and intraventricular hemorrhage (IVH) in Supplementary Table S1. We sub-classified SAH into minor and major, according to a modified Fisher Scale [8]. In case of the coexistence of more than one type of hemorrhage, we selected the dominant one.

We finally grouped HI and minor SAH as “minor HT” and PH, rPH, major SAH, and IVH as “major HT”. The primary outcome was the time between the start of the first recanalization treatment and the start of antithrombotic or anticoagulant secondary stroke prevention. Secondary outcomes were recurrent ischemic and hemorrhagic stroke as well as death all within three months from the index stroke event. All recurrent strokes of the ICH type were assessed as having a de novo symptomatic ICH as the main diagnosis. The manuscript was written following the STROBE checklist.

### Statistical Analysis

Median and interquartile ranges were reported for continuous variables, frequencies and percentages for categorical variables. Descriptive statistics were used to illustrate the baseline demographic and clinical characteristics of this study’s population between hemorrhagic and non-hemorrhagic patients, patients suffering from none, minor and major HT, and between hospitals. Differences between groups were compared using Pearson’s chi-squared test for categorical variables and the Wilcoxon rank-sum (2 groups) or Kruskal–Wallis (>2 groups) tests for continuous variables. To control for selection bias and baseline differences, additional analysis was performed using propensity score matching. Matching was performed on patients who experienced hemorrhage vs. non-hemorrhage. A logistic regression model was fitted for the patient’s status (hemorrhagic vs. non-hemorrhagic), while pairing was executed with a 1:1 ratio, the nearest neighbor method and a 0.2 width of caliper [9]. The variables previous antithrombotic therapy, atrial fibrillation, baseline NIHSS and treatment (IVT vs. EVT vs. IVT + EVT) were introduced into the propensity score model. Matched groups were summarized using descriptive statistics and covariate balance after matching was confirmed by standardized mean differences as percentages [10]. Lastly, propensity-score-weighted Kaplan–Meier curves were created to illustrate the rates of receiving secondary prevention treatment stratified by hemorrhagic type. Differences between cumulative incidence functions of the three groups were compared using the log-rank test. Analyses were performed using Stata Statistical Software: Release 15. (Stata Corp. 2017. College Station, TX, USA). All *p*-values were two-sided, and a *p*-value < 0.05 was considered statistically significant.

## 3. Results

We enrolled 862 patients (311 patients Rome, 551 Stockholm), 416 females and 446 males. The median age was 72 years. A total of 345 (40.0%) patients received IVT alone, 266 (30.8%) combined IVT and EVT, and 251 (29.1%) EVT alone. 

### 3.1. No HT vs. HT: Baseline Characteristics before and after Matching

Table 1 shows a comparison between the baseline characteristics of patients with and without post-revascularization HT before matching.

Before-matching patients with HT had more AF (47% vs. 32%), a higher median baseline NIHSS (17 vs. 11) and had more often received EVT (77% vs. 55%), while having similar age, sex and risk factor distribution (Table 1). 

Looking at baseline characteristics after matching, the groups were well balanced in AF, NIHSS, distribution of reperfusion treatments, baseline HAS-BLED and CHA₂DS₂-VASc scores, previous antithrombotic therapy and other parameters (Supplementary Table S2). Patient characteristics and outcomes at each center are summarized in Supplementary Table S3. 

### 3.2. No HT vs. HT: Outcomes before and after Matching

Table 2 shows a comparison of outcomes between patients with and without HT before and after matching. 

Before matching, in the HT group, there were more patients who did not start any antithrombotic treatment within 3 months (14 vs. 11 patients, in percentage 6.7% vs. 1.7%, respectively) compared to the no HT group. Moreover, before matching, the delay of secondary prevention was slightly higher in patients with HT (28 h vs. 24 h, with interquartile ranges 22–63 vs. 22–31, respectively). Finally, more patients with HT died within three months compared to the no HT group before matching (53 vs. 30 patients, in percentage 14.4% vs. 8.1%, respectively). 

After matching, the proportion of patients who did not start antithrombotics (14 patients vs. 4 patients, 6.8% vs. 2%, respectively) and those with a delay in their start (28 vs. 24 h, with interquartile ranges 18.5–36 vs. 22–63, respectively) remained higher in the HT group. In the matched HT group, 75% had minor hemorrhage (HI or minor SAH), and 25% had major hemorrhage (PH or major SAH/IVH).

### 3.3. No Hemorrhage vs. Minor Hemorrhage vs. Major Hemorrhage: Characteristics and Outcomes after Matching

Table 3 shows the clinical characteristics and the outcomes in patients without hemorrhage vs. minor HT vs. major HT after matching.

Supplementary Table S4 shows the differences regarding both baseline characteristics and outcomes between the corresponding unmatched groups.

**Figure 1:**
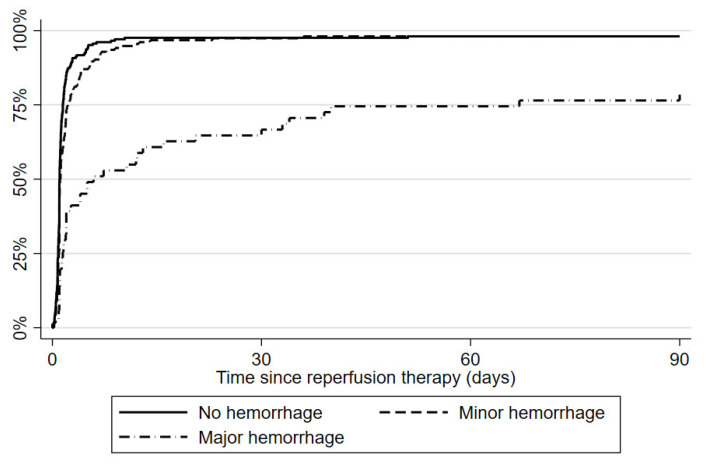
Proportion of patients starting secondary prevention over 3 months.

Patients with minor HT did not have any significant delay in the start of antithrombotics compared to patients with no hemorrhage (median 26 vs. 24 h). Figure 1 shows that the start of antithrombotics was very similar in patients without hemorrhage and those with minor HT.

A total of 1.9% of patients with minor HT and 2% of patients without hemorrhage did not start any antithrombotic therapy within 3 months. Nevertheless, patients with minor HT had stroke recurrence (both ischemic and hemorrhagic) and death rates similar to those of patients without hemorrhage. Regarding baseline characteristics, we found no significant differences between minor and no hemorrhage groups, except for female sex (59% vs. 44%).

In contrast, patients with major HT started antithrombotics significantly later compared to patients with no hemorrhage or minor HT (median time 39 h). The IQR of the starting time was large in the major HT group, from 21 h to over 10 days. Moreover, 21.6% of patients with major HT did not start any secondary prevention therapy within 3 months, as shown in Figure 1. Ischemic stroke recurrence at 3 months was similar to that observed in minor HT and no hemorrhage groups, while hemorrhagic stroke was more frequent (3.9%). This brought the total recurrence rate in major HT to 7.8%. Finally, three-month mortality was significantly higher in this group (25.5%). Patients with major HT did not differ significantly from the other groups concerning baseline characteristics, except for previous use of dual antiplatelet therapy. 

## 4. Discussion

In the routine clinical practice of two comprehensive stroke centers in Sweden and Italy, the starting time of antithrombotic secondary prevention among reperfusion-treated stroke patients was similar. The majority of hemorrhages after IVT and EVT were minor (75%). We found that a delay in starting antithrombotics among patients with post-treatment hemorrhage was only seen in cases with parenchymal hematomas and major SAH/IVH. Conversely, patients with purely petechial hemorrhagic infarct transformation or minor SAH were started on antithrombotics just as early as patients without any hemorrhage (median: 26 vs. 24 h after IVT or EVT). The occurrence of safety outcomes, such as early neurological deterioration, stroke recurrence and death within three months, were all very similar between the minor and no hemorrhage groups. 

The finding that minor hemorrhage, classified as HI, is of no significant prognostic importance, has been reported previously in the IVT literature [11,12]. We found it noteworthy that minor hemorrhagic findings on follow-up imaging after 24 h did not influence the start of antithrombotic drugs to any significant degree and that such patients did not tend to deteriorate or recur with new hemorrhagic stroke after the start of antithrombotics. These findings are in line with a previous detailed study by Kim et al. on patients with both spontaneous and post-revascularization HI [7].

Another noteworthy finding was that patients with major hemorrhage after IVT or EVT, despite having a longer delay in starting antithrombotic treatment, did not have a significantly higher frequency of ischemic stroke recurrence compared to the other two groups. While somewhat reassuring, this must be interpreted bearing in mind that this is confounded by a higher frequency of death within 3 months in this group. While deaths were not caused by recurrent stroke, they may have censored the population, as these patients might have had an elevated recurrence risk had they remained alive. Similar caveats apply also to the interpretation of the rate of hemorrhagic stroke recurrence in major HT patients at 3.9%. 

The major HT group differed significantly from the minor HT and no hemorrhage groups. After major HT, almost 22% of patients did not start any antithrombotic drug, and, when started, the delay was significantly longer with a very wide interval. Despite these differences, ischemic stroke recurrence was low and did not differ significantly between groups. While subject to potential sources of confounding, this may be somewhat reassuring, as it could indicate that delaying antithrombotics in major HT patients to avoid hematoma expansion or re-bleeding might be associated with a limited risk of clinically apparent ischemic recurrence.

While this study provides evidence on secondary prevention after post-revascularization HT, therapeutic choices after spontaneous HT remain a matter of debate. The rationale behind only selecting patients treated with reperfusion therapies for this study was twofold: such patients nearly universally have follow-up imaging at relatively standardized time points, and they have also been shown to have a higher occurrence of hemorrhagic transformation compared to patients without IVT or EVT [13].

Although propensity score matching mitigates confounding by baseline differences and by indication, strengthening the results of our study, we would like to point out some limitations. Our dataset did not include some variables, which could be important for clinicians’ assessment of the risk for hemorrhagic transformation (and thus could influence the start of treatment)—e.g., blood pressure and blood glucose levels, etiology, and the size of infarct on imaging at 24 h. This precluded us from matching the groups for these potentially relevant factors. Further, we cannot exclude some degree of confounding by unmeasured center-specific care parameters or population differences. However, our findings on HT rates and on predictors for the occurrence of post-revascularization HT are consistent with previous studies [3,4,5,14,15]. Furthermore, merging data from two large centers in different countries reduces the influence of specific geographical and national factors. These considerations and the consistency with the literature lead us to believe that our sample is representative of the general population of patients with post-revascularization HT and strengthens the reliability of our findings. Moreover, center differences would be unlikely to have a major influence on the main patterns of our findings, as the median antithrombotic starting time between our hospitals differed by only 5 h (Supplementary Table S4). Another potential limitation is linked to the different timeframes of patient recruitment in the two centers. Meanwhile, the criteria for IVT or EVT treatment did not differ significantly between the hospitals (Supplementary Methods). Finally, the low incidence of major HT and stroke recurrence likely limited our power to detect statistically significant differences between major HT and no hemorrhage/minor HT groups. This supports the need for large international registries to address this issue. 

## 5. Conclusions

The presence of hemorrhagic findings on follow-up imaging influences the timing of antithrombotic secondary prevention after stroke thrombolysis and thrombectomy. Our results show that patients with major hemorrhagic findings on follow-up imaging start secondary prevention later or not at all in the first three months. While our results did not show a significantly elevated risk of early ischemic or hemorrhagic stroke recurrence in this group, limited case numbers and potential confounding warrant caution, and our results need to be verified in larger studies. Meanwhile, patients with only petechial or minor subarachnoid hemorrhage may start antithrombotic therapy equally early as those with no hemorrhage with similar safety. 

## Figures and Tables

**Table 1:** Comparison of baseline characteristics between no HT and HT groups before matching.

Characteristics	Before Matching		
	No HT (n = 653)	HT (n = 209)	*p*-Value
Female	319 (48.9%)	97 (46.4%)	0.54
Age	72 (62–80)	72 (62–80)	0.81
Prev. Antithromb. Therapy	265 (40.6%)	86 (41.1%)	0.88
Prev. Antithromb. Therapy (Detailed)			
None	388 (59.4%)	123 (58.9%)	
Single antiplatelet	180 (27.6%)	46 (22.0%)	
Dual antiplatelet	12 (1.8%)	11 (5.3%)	
Oral anticoagulant (VKA/DOAC)	70 (10.7%)	26(12.4%)	
Other	3 (0.5%)	3 (1.4%)	
AF	211 (32.3%)	99 (47.4%)	<0.001
Hypertension	427 (65.4%)	142 (67.9%)	0.50
Diabetes	100 (15.3%)	40 (19.1%)	0.19
Smoking	125 (19.1%)	39 (18.7%)	0.88
Previous stroke	72 (11.0%)	27 (12.9%)	0.46
CHA2DS2-VASc	5 (4–6)	5.00 (3–6)	0.64
HAS-BLED	3 (2–3)	3 (2–4)	0.56
NIHSS	11 (6–17)	17 (12–21)	<0.001
Treatment			<0.001
IVT alone	297 (45.4%)	48 (23.0%)	
EVT alone	176 (27.0%)	75 (35.9%)	
EVT + IVT	180 (27.6%)	86 (41.1%)	
Onset- or LKW-to-IVT (min)	125 (90–180)	105 (85–150)	0.011
Onset or LKW to artery puncture (min)	240 (180–355)	242.5 (177–355)	0.85
Extracranial bleeding before AT start	24 (3.7%)	8 (3.8%)	0.92
Neurol. deterioration within 24h	38 (5.8%)	28 (13.4%)	<0.001
HT subtypes			
HI1		69 (33.0%)	
HI2		63 (30.1%)	
PH1		20 (9.6%)	
PH2		16 (7.7%)	
rPH		12 (5.7%)	
Minor SAH		26 (12.4%)	
Major SAH/IVH		3 (1.4%)	
HT			
None	653 (100.0%)		
HI/Minor SAH		158 (75.6%)	
PH/Major SAH/IVH		51 (24.4%)	

HT: hemorrhagic transformation; VKA: Vitamin K Antagonists; DOAC: Direct Oral Anticoagulants; AF: atrial fibrillation; IVT: intravenous thrombolysis; EVT: endovascular thrombectomy; LKW: Last Known Well; AT: antithrombotic therapy; HI: hemorrhagic infarction; PH: parenchymal hematoma; rPH: remote parenchymal hematoma; SAH: subarachnoid hemorrhage; IVH: intraventricular hemorrhage. Values denote medians with interquartile range (IQR) or numbers with percentage.

**Table 2:** Comparison of outcomes between no HT group and HT group before and after matching.

	Before Matching			After Matching		
	No HT (n = 653)	HT (n = 209)	*p*-Value	No HT (n = 205)	HT (n = 205)	*p*-Value
Start of antithrombotic (hours from reperf. therapy)	24 (22–31)	28 (22–63)	<0.001	24 (18.5–36)	28.00 (22–63)	0.002
No antithrombotics in the first 3 months	11 (1.7%)	14 (6.7%)	<0.001	4 (2.0%)	14 (6.8%)	0.016
First antithrombotic agent						
Antiplatelet	532 (83.5%)	140 (73.3%)		159 (79.5%)	139 (73.2%)	
Oral anticoagulant	37 (5.8%)	15 (7.9%)		13 (6.5%)	15 (7.9%)	
LMWH	68 (10.7%)	36 (18.8%)		28 (14.0%)	36 (18.9%)	
Stroke recurrence within 3 months			0.058			0.19
Ischemic	18 (2.8%)	5 (2.4%)		7 (3.4%)	5 (2.4%)	
Hemorrhagic	1 (0.2%)	3 (1.4%)		0 (0.0%)	3 (1.5%)	
Time of stroke recurrence after index event (days)	10 (4–30)	10 (6–28)	0.72	6.5 (4–21)	10 (6–28)	0.18
Death within 3 months	53 (8.1%)	30 (14.4%)	0.008	19 (9.3%)	30 (14.6%)	0.098

HT: hemorrhagic transformation, LMWH: Low Molecular Weight Heparin. Values denote medians with interquartile range (IQR) or numbers with percentage.

**Table 3:** Comparison of no hemorrhage group vs. minor hemorrhage group vs. major hemorrhage group regarding baseline characteristics and outcomes after matching.

Characteristics of Matched Patients	No Hemorrhage	HI/Minor SAH	PH/Major SAH	*p*-Value
	n = 205	n = 154	n = 51	
Female	121 (59.0%)	68 (44.2%)	27 (52.9%)	0.020
Age	74 (65–82)	71 (61–81)	73 (64–82)	0.46
Previous antithrombotic therapy	88 (42.9%)	63 (40.9%)	22 (43.1%)	0.92
Previous antithrombotic therapy (detailed)				
None	117 (57.1%)	91 (59.1%)	29 (56.9%)	
Single antiplatelet therapy	55 (26.8%)	33 (21.4%)	13 (25.5%)	
Double antiplatelet therapy	3 (1.5%)	5 (3.2%)	5 (9.8%)	
Oral anticoagulant (VKA/DOAC)	30 (14.6%)	22 (14.3%)	4 (7.8%)	
Other	0 (0.0%)	3 (1.9%)	0 (0.0%)	
AF	98 (47.8%)	77 (50.0%)	21 (41.2%)	0.55
Hypertension	133 (64.9%)	104 (67.5%)	36 (70.6%)	0.71
Diabetes	33 (16.1%)	29 (18.8%)	10 (19.6%)	0.73
Smoking	23 (11.2%)	29 (18.8%)	8 (15.7%)	0.13
Previous stroke	23 (11.2%)	17 (11.0%)	9 (17.6%)	0.41
CHA2DS2-VASc	5 (4–6)	5 (3–6)	5 (4–6)	0.14
HAS-BLED	3 (2–4)	3 (2–3)	3 (3–4)	0.26
NIHSS	17(13–20)	17 (13–21)	17 (10–21)	0.54
Treatment				
IVT alone	50 (24.4%)	28 (18.2%)	19 (37.3%)	
EVT alone	66 (32.2%)	59 (38.3%)	16 (31.4%)	
EVT + IVT	89 (43.4%)	67 (43.5%)	16 (31.4%)	
Onset- or LKW-to-IVT (min)	116 (80–147)	105 (85–145)	118.5 (87–180)	0.70
Onset or LKW to artery puncture (min)	222 (177–330)	257 (185–360)	220 (170–294)	0.25
Extracranial bleeding before AT start	8 (3.9%)	5 (3.2%)	3 (5.9%)	0.70
Neurological deterioration within 24h	9 (4.4%)	10 (6.5%)	17 (33.3%)	<0.001
HT subtypes				
HI1		67 (43.5%)		
HI2		62 (40.3%)		
PH1			20 (39.2%)	
PH2			16 (31.4%)	
rPH			12 (23.5%)	
Minor SAH/IVH		25 (16.2%)		
Major SAH/IVH			3 (5.9%)	
Start of antithrombotic (hours from reperf. therapy)	24 (18.5–36)	26 (22–48)	39 (21–256)	0.003
No initiation of antithrombotics in the first 3 months	4 (2.0%)	3 (1.9%)	11 (21.6%)	<0.001
First antithrombotic agent				
Antiplatelet therapy	159 (79.5%)	115 (76.7%)	24 (60.0%)	
Oral anticoagulants	13 (6.5%)	9 (6.0%)	6 (15.0%)	
LMWH	28 (14.0%)	26 (17.3%)	10 (25.0%)	
Stroke recurrence within 3 months				0.22
Ischemic	7 (3.4%)	3 (1.9%)	2 (3.9%)	
Hemorrhagic	0 (0%)	1 (0.6%)	2 (3.9%)	
Time of stroke recurrence after index event (days)	6.5 (4–21)	12 (6–28)	9 (5.5–27)	0.41
Death within 3 months	19 (9.3%)	17 (11.0%)	13 (25.5%)	0.006

HI: hemorrhagic infarction; SAH: subarachnoid hemorrhage; PH: parenchymal hematoma; VKA: Vitamin K Antagonists; DOAC: Direct Oral Anticoagulants; AF: atrial fibrillation; HT: hemorrhagic transformation; IVT: intravenous thrombolysis; EVT: endovascular thrombectomy; LKW: Last Known Well; AT: antithrombotic therapy; rPH: remote parenchymal hematoma; IVH: intraventricular hemorrhage; LMWH: Low Molecular Weight Heparin.

## Data Availability

The data presented in this study are available on request from the corresponding author.

## Supplementary Materials

The following supporting information can be downloaded at: https://www.mdpi.com/article/10.3390/jcm12082771/s1. Supplementary Methods: Internal Guidelines for each Center; Supplementary Table S1: Classification of hemorrhagic transformation; Supplementary Table S2: Patients’ baseline characteristics after matching. No hemorrhage vs. hemorrhage; Supplementary Table S3: Patients’ baseline characteristics at each center; Supplementary Table S4: Differences between unmatched patients with no hemorrhage, minor HT and major HT.
