# Supplementary material for: Timing of Antithrombotic Secondary Prevention in Patients with Intracranial Hemorrhage after Stroke Thrombolysis and Thrombectomy
Source: J Clin Med. 2023 Apr 7;12(8):2771. doi: 10.3390/jcm12082771 (PMC10145350; doi:10.3390/jcm12082771)
Supplement: Supplementary file 1 [file jcm-12-02771-s001.zip › jcm-2211690-supplementary.pdf]

Supplementary Materials

Supplementary Methods: Internal guidelines for each Centre

Internal guidelines for IVT (respectively 2016-2019 and 2013-2016) – Fondazione Policlinico Universitario A. Gemelli and Karolinska University Hospital.

- > 18 yo
- Symptoms onset < 4.5 hours
- NIHSS ≥ 5 (< 5 if disabling symptoms, e.g. hemianopia, aphasia, etc)
- Early ischemic changes < 1/3 of middle cerebral artery territory at brain CT
- Platelet count > 100000, INR < 1.7, blood glucose < 200 mg/dL, BP < 185/110 mmHg
- Exclude patients taking LMWH at therapeutic dosage or patients on DOACS
- Exclude patients at high risk of systemic/intracranial hemorrhage

Internal guidelines for EVT 2016-2019 – Fondazione Policlinico Universitario A. Gemelli

- Symptoms onset < 6 hours (when <4.5 hours, rTPA first, if not contraindicated)
- Large artery occlusion (internal carotid artery, middle cerebral artery M1, tandem occlusion, anterior cerebral artery, basilar artery)
- ASPECTS ≥ 6

Internal guidelines for EVT 2013-2016 – Karolinska University Hospital

- Symptom onset < 8 hours (when <4.5 hours, IVT first, if not contraindicated)
- Large artery occlusion (internal carotid artery, middle cerebral artery M1 or M2, tandem occlusion, basilar artery)
- NIHSS ≥ 6 (exception for cases with NIHSS <6 if aphasia present)
- CT infarct signs exceeding 1/3 of the MCA territory or with extensive brain stem involvement
- Exclude patients with co-morbidities limiting expected pre-stroke life span to < 3 months

Supplementary Tables

Supplementary Table S1. Classification of Hemorrhagic transformation.

Type	Definition	Dichotomic classification
HI1	Non-confluent small petechiae within the infarcted area, without space-occupying effect	Minor
HI2	Confluent petechiae within the infarcted area, no space-occupying effect	Minor
PH1	Intralesional confluent hematoma <30% of the infarcted area, with no or mild space occupying-effect	Major
PH2	Intralesional confluent hematoma >30% of the infarcted area, with significant space-occupying effect	Major
rPH1	Extralesional hematoma, remote from the acute infarct, with mild space-occupying effect	Major
rPH2	Extralesional hematoma, remote from the acute infarct, with significant space-occupying effect	Major
Minor SAH	Subarachnoid Hemorrhage, modified Fisher Scale 1	Minor
Major SAH	Subarachnoid Hemorrhage, modified Fisher Scale 2-4	Major
IVH	Intraventricular Hemorrhage	Major

HI: hemorrhagic infarction, PH: parenchymal hematoma, rPH: remote parenchymal hematoma, SAH: subarachnoid hemorrhage, IVH: intraventricular hemorrhage

Supplementary Table S2: Patients baseline characteristics after matching. No Hemorrhage vs Hemorrhage

Characteristics	After Matching		
	No Hemorrhage	Hemorrhage	p-value
	**N=205**	**N=205**	
Female	121 (59.0%)	95 (46.3%)	0.01
Age	74 (65-82)	72 (62-81)	0.24
Prev. Antithromb. Therapy	88 (42.9%)	85 (41.5%)	0.76
Prev. Antithromb. Therapy (Detailed)			
None	117 (57.1%)	120 (58.5%)	
Single antiplatelet	55 (26.8%)	46 (22.4%)	
Dual antiplatelet	3 (1.5%)	10 (4.9%)	
Oral anticoagulant (VKA/DOAC)	30 (14.6%)	26 (12.7%)	
Other	0 (0.0%)	3 (1.5%)	
AF	98 (47.8%)	98 (47.8%)	1.00
Hypertension	133 (64.9%)	140 (68.3%)	0.46
Diabetes	33 (16.1%)	39 (19.0%)	0.44
Smoking	23 (11.2%)	37 (18.0%)	0.05
Previous stroke	23 (11.2%)	26 (12.7%)	0.65
CHA_2DS_2-VASc	5 (4-6)	5 (3-6)	0.33
HAS-BLED (baseline)	3 (2-4)	3.00 (2-4)	0.28
NIHSS (baseline)	17 (13-20)	17 (12-21)	0.76
Treatment			
IVT alone	50 (24.4%)	47 (22.9%)	
EVT alone	66 (32.2%)	75 (36.6%)	
EVT+IVT	89 (43.4%)	83 (40.5%)	
Onset-to-IVT (or LKW-to-IVT) (min)	116 (80-147)	110 (85-150)	0.96
Onset-to-groin puncture (or LKW-to-groin puncture) (min)	222 (177-330)	240 (177-355)	0.32
Extracranial bleeding before AT start	8 (3.9%)	8 (3.9%)	1.00
Neurol. deterioration within 24h	9 (4.4%)	27 (13.2%)	0.002
HT subtypes			
HI1		67 (32.7%)	
HI2		62 (30.2%)	
PH1		20 (9.8%)	
PH2		16 (7.8%)	
rPH		12 (5.9%)	
Minor SAH		25 (12.2%)	
Major SAH/IVH		3 (1.5%)	
HT			
None	205 (100.0%)		
HI/Minor SAH		154 (75.1%)	
PH/MajorSAH/IVH		51 (24.9%)	

VKA: Vitamin K Antagonists; DOAC: Direct Oral Anticoagulants; AF: Atrial Fibrillation; HT: Hemorrhagic Transformation; IVT: Intravenous thrombolysis; EVT: Endovascular Thrombectomy; LKW: Last Known Well; AT: Antithrombotic Therapy; SAH: Subarachnoid Hemorrhage; IVH: Intraventricular Hemorrhage.

Supplementary Table S3: Patients baseline characteristics at each Center (FPG: Fondazione Policlinico Universitario A. Gemelli IRCCS. Karolinska: Karolinska University Hospital)

	**IVT**		**EVT**		**IVT + EVT**		**IVT, IVT + EVT, EVT**			**Pooled**
Centre	FPG (166/311)	Karolinska (179/551)	FPG (65/311)	Karolinska (186/551)	FPG (80/311)	Karolinska (186/551)	FPG (311)	Karolinska (551)	p	FPG + Karolinska (862)
**Males/Females**	82/84	95/84	26/40	103/83	38/42	103/83	146/165	300/251	0.03	446/416
**Age (median, range)**	77,5 (25-100)	72 (23-97)	77 (48-93)	70 (6-89)	74 (28-93)	68 (20-97)	76 (25-100)	70 (6-97)	<0.001	72 (6-100)
**Patients already on antithrombotic treatment**	84 (51%)	57 (32%)	39 (60%)	84 (45%)	30 (38%)	56 (30%)	153 (49%)	185 (36%)	<0.001	350 (41%)
Single antiplatelet therapy	70	47	11	39	18	41	99	127		226
Double antiplatelet therapy	9	3	2	1	5	3	16	7		23
VKA	5	4	18	31	6	10	29	45		74
DOACs	0	2	6	11	1	1	7	14		21
Others (antiplatelet+VKA…)	0	1	2	2	0	1	2	4		16
**Risk factors**										
AF (previous or new diagnosis)	44 (27%)	51 (29%)	35 (53%)	82 (44%)	29 (36%)	69 (37%)	108 (35%)	202 (37%)	n.s.	310 (36%)
Hypertension	140 (87%)	114 (64%)	32 (48%)	108 (58%)	56 (70%)	99 (53%)	248 (80%)	321 (58%)	<0.001	569 (66%)
Diabetes	35 (21%)	25 (14%)	12 (18%)	32 (17%)	15 (19%)	21 (11%)	62 (20%)	78 (14%)	0.03	140 (16%)
Smoke (current or previous)	36 (22%)	23 (13%)	13 (20%)	31(17%)	29 (36%)	32 (17%)	78 (25%)	86 (16%)	0.001	164 (19%)
Previous stroke	23 (14%)	20 (11%)	6 (9%)	25 (13%)	8 (10%)	17 (9%)	37 (12%)	62 (11%)	n.s.	99 (11%)
CHA_2DS_2-VASc (median, range)	5 (2-8)	5 (2-8)	6 (2-8)	4 (2-8)	5 (2-9)	4 (2-8)	5 (2-9)	4 (2-8)	<0.001	5 (2-9)
HAS-BLED (median, range)	3 (2-5)	3 (1-6)	3 (2-6)	3 (2-6)	3 (1-6)	3 (1-5)	3 (1-6)	3 (1-6)	<0.001	3 (1-6)

IVT only							166 (53%)	179 (33%)	<0.001	
EVT only							65 (21%)	186 (34%)	<0.001	
IVT + EVT							80 (26%)	186 (34%)	0.01	
Permanent stenting placement							15 (5%)	81 (15%)	<0.001	
Secondary transfer							46 (15%)	282 (76%)	<0.001	
**Onset NIHSS (median, range)**	9 (1-25)	7 (0-28)	18 (4-33)	13.5 (0-37)	18 (1-30)	15.5 (1-37)	13 (1-33)	12 (0-37)	n.s.	13 (0-37)
**Onset-to-needle, min (median, range)**	145 (30-360)	112 (20-790)	-	-	142.5 (50-300)	95 (35-540)	145 (30-360)	104 (20-790)	<0.001	120 (20-790)
**Onset or LKW-to-artery, min (median and range)**	-	-	315 (60-140)	285 (55-6120)	221 (18-420)	225 (74-1520)	240 (60-1140)	240 (55-6120)	n.s.	240 (55-6120)
**Major extracranial bleeding within first 24 h after revascularization**	2 (1%)	8 (4%)	1 (2%)	9 (5%)	1 (1%)	11 (6%)	4 (1%)	28 (5%)	0.05	32 (4%)
**Post-treatment intracranial haemorrhage total**	19 (11%)	29 (16%)	25 (38%)	50 (27%)	30 (38%)	56 (30%)	74 (24%)	135 (25%)	n.s.	209 (24%)
HI1	7	10	6	16	8	22	21	48		69
HI2	7	2	13	16	8	17	28	35		63
PH1	2	3	3	7	2	3	7	13		20
PH2	1	4	2	2	4	3	7	9		16
rPH	1	7	0	0	2	2	3	9		12
Minor SAH/IVH	0	3	0	8	6	9	6	20		26
Major SAH/IVH	1	0	1	1	0	0	2	1		3
**Start of secondary prevention, hours, hours (median and range)**	24 (18-720)	28 (5-2880)	24 (0-72)	28 (1-224)	24 (0-936)	30 (4-864)	24 (0-936)	29 (1-2880)	<0.001	24 (0-2880)
**Stroke recurrence at three months**	6 (4%)	8 (4%)	0 (0%)	6 (3%)	0 (0%)	5 (3%)	6 (2%)	19 (3%)	n.s.	25 (3%)
**Death at three months**	7 (4%)	16 (9%)	16 (24%)	16 (9%)	16 (20%)	12 (6%)	39 (13%)	44 (8%)	0.03	83 (10%)

**Days from index event (median, range)**	21 (5-85)	6 (2-42)	14 (4-70)	4 (1-9)	21 (4-65)	3 (1-19)	16.5 (4-85)	4 (1-42)	<0.001	10 (1-85)

IVT: Intravenous thrombolysis; EVT: Endovascular Thrombectomy; FPG: Fondazione Policlinico Gemelli; VKA: Vitamine K Antagonists; DOAC: Direct Oral Anticoagulants; AF: Atrial Fibrillation; HI: Hemorrhagic Infarction; PH: Parenchymal Hematoma; rPH: Remote Parenchymal Hematoma; SAH: Subarachnoid Hemorrhage; IVH: Intraventricular Hemorrhage.

Supplementary Table S4: Differences between unmatched patients with no-hemorrhage vs minor HT vs major HT.

	**none N=653**	**HI/Minor SAH N=158**	**PH/Major SAH N=51**	**p-value**
Female	319 (48.9%)	70 (44.3%)	27 (52.9%)	0.47
Age	72.00 (62.00-80.00)	71.00 (61.00-80.00)	73.00 (64.00-82.00)	0.84
Previous Antithrombotic Therapy	265 (40.6%)	64 (40.5%)	22 (43.1%)	0.94
Previous Antithrombotic Therapy (Detailed)				0.009
none	388 (59.4%)	94 (59.5%)	29 (56.9%)	
Single antiplatelet therapy	180 (27.6%)	33 (20.9%)	13 (25.5%)	
Double antiplatelet therapy	12 (1.8%)	6 (3.8%)	5 (9.8%)	
Oral anticoagulant (VKA/DOAC)	70 (10.7%)	22 (13.9%)	4 (7.8%)	
Other	3 (0.5%)	3 (1.9%)	0 (0.0%)	
AF	211 (32.3%)	78 (49.4%)	21 (41.2%)	<0.001
Hypertension	427 (65.4%)	106 (67.1%)	36 (70.6%)	0.72
Diabetes	100 (15.3%)	30 (19.0%)	10 (19.6%)	0.42
Smoking	125 (19.1%)	31 (19.6%)	8 (15.7%)	0.81
Previous stroke	72 (11.0%)	18 (11.4%)	9 (17.6%)	0.36
CHA_2DS_2-VASc	5.00 (4.00-6.00)	5.00 (3.00-6.00)	5.00 (4.00-6.00)	0.16
HAS-BLED	3.00 (2.00-3.00)	3.00 (2.00-3.00)	3.00 (3.00-4.00)	0.38
NIHSS	11.00 (6.00-17.00)	17.00 (13.00-21.00)	17.00 (10.00-21.00)	<0.001

Treatment				<0.001
IVT	297 (45.5%)	29 (18.4%)	19 (37.3%)	
EVT	176 (27.0%)	59 (37.3%)	16 (31.4%)	
EVT+IVT	180 (27.6%)	70 (44.3%)	16 (31.4%)	
Onset or LKW-to-IVT (min)	125.00 (90.00-180.00)	105.00 (85.00-140.00)	118.50 (87.00-180.00)	0.021
Onset or LKW-to-groin puncture (min)	240.00 (180.00-355.50)	260.00 (185.50-362.50)	220.00 (170.00-294.00)	0.38
Extracranial bleeding before AT start	24 (3.7%)	5 (3.2%)	3 (5.9%)	0.67
Neurological deterioration within 24h	38 (5.8%)	11 (7.0%)	17 (33.3%)	<0.001
HT subtypes				<0.001
HI1		69 (43.7%)		
HI2		63 (39.9%)		
PH1			20 (39.2%)	
PH2			16 (31.4%)	
rPH			12 (23.5%)	
Minor SAH/IVH		26 (16.5%)		
Major SAH/IVH			3 (5.9%)	
No initiation of antithrombotics in the first 3 month	11 (1.7%)	3 (1.9%)	11 (21.6%)	<0.001
START of antithrombotic (hours from reperf therapy)	24.00 (22.00-31.00)	26.00 (22.00-50.00)	39.00 (21.00-256.00)	<0.001
First antithrombotic agent				0.002
Antiplatelet therapy	532 (83.5%)	116 (76.8%)	24 (60.0%)	
Oral anticoagulants	37 (5.8%)	9 (6.0%)	6 (15.0%)	
LMWH	68 (10.7%)	26 (17.2%)	10 (25.0%)	
Stroke recurrence within 3 months				0.004
Ischemic	18 (2.8%)	3 (1.9%)	2 (3.9%)	
Hemorrhagic	1 (0.2%)	1 (0.6%)	2 (3.9%)	
Death within 3 months	53 (8.1%)	17 (10.8%)	13 (25.5%)	<0.001
Time of stroke recurrence after index event (days)	10.00 (4.00-30.00)	12.00 (6.00-28.00)	9.00 (5.50-27.00)	0.93

Data are presented as median (IQR) for continuous measures, and n (%) for categorical measures.

HI: Hemorrhagic Infarction; PH: Parenchymal Hematoma; SAH: Subarachnoid Hemorrhage; VKA: Vitamin K Antagonists; DOAC: Direct Oral Anticoagulants; AF: Atrial Fibrillation; HT: Hemorrhagic Transformation; IVT: Intravenous thrombolysis; EVT: Endovascular Thrombectomy; LKW: Last Known Well; AT: Antithrombotic Therapy; IVH: Intraventricular Hemorrhage; SEC PREV: Secondary Prevention.
